# Gleanings

**Published:** 1897-08

**Authors:** 


### GLEANINGS.

Vermont is considering a proposition made by Dr. Seward Webb, the famous hackney-breeder, to bond the State in raising a sufficient sum of money to build improved roads through the three valleys traversing the length of the State from north to south. It will prove the best investment the State has ever made, making her fertile hills and valleys so accessible through intersecting good roads that she may well hope to win back a goodly portion of the thirty or more millions of dollars that her farms alone have shrunk in the last twenty-five years.

Picric acid in burns and scalds may be used as a powder sprinkled on the affected surfaces, or compresses saturated with a solution of the acid. Bathing the parts with a solution every few hours gives great relief where the pain is marked. Two drachms to a quart of water make a saturated solution; an ounce of alcohol to a quart of water increases the solubility; vaseline on the hands will prevent staining of the latter when using. Blisters should be punctured, not incised, to promote healing more rapidly.—*Therapeutic Gazette.*

The Attorney-General of New York State has decided that the State Board of Health has no power to prohibit the importation of cattle into the State, to quarantine them, or to require the tuberculin-test to be applied to such cattle; holding that such powers must be legislative in character.

The dipping-vats for sheep in the larger stock-yards promise fruitful results from the losses entailed in this direction, and proper disinfection of the cars will be a long step toward the better control of "scab."

The sheep-dipping-vat at Chicago is some ninety-four feet long, the tobacco solution being used is maintained at a temperature of 120°. About one thousand sheep can be dipped hourly. A charge of three cents per head is made, and all sheep for export must be dipped and carry with them a certificate of the same.

Twenty thousand sheep were recently condemned at the port of entry, Marseilles, France, suffering from a disease unfitting them for food.

There are many applicants for the veterinary chair in the Kansas Agricultural College recently made vacant by the action of a Populistic Board of Regents.

Hooven, or bloating, among sheep, when fed too much rape, is said to yield readily to the use of ammonia spirits given by drench in water. The use of the trocar and canula in very great distention yields prompt results.

A practical hog-breeder recommends the actual cautery along the loins and back for those cases of loss of power of the hind-quarters and dragging of these parts.

The *Veterinary Record* quotes Herr Gmeiner, veterinary surgeon of Coburg, as suggesting the following treatment for grease: Free the limb of all diseased products by means of hot water thrown on with force by aid of a syringe, then dry the skin with cotton-wool or tow; then apply pledgets soaked with ordinary turpentine in regular layers, and maintain in position with a bandage, repeating daily, or every other day, as indicated, washing so long as the uncleanliness exists.

**An excellent programme greets you Sept. 7th, 8th, and 9th.**

The death of a valuable horse and the illness of a number of other horses and mules from eating improperly cured pea-vine hay is reported by Dr. W. H. Dalrymple, of Louisiana.

An English cattle-owner hopes to see the day when it wlil be compulsory to brand cows that have aborted their calves.

A correspondent reports a capped elbow with extensive granulations very much aided in recovery by being placed in slings after being operated upon.

Ascites in a spaniel dog showing no improvement after tapping and internal treatment, an opening in the abdominal cavity was made, a weak solution of carbolic acid and glycerine used for washing out the cavity, with drainage-tube inserted, but death followed some five days later.

**We will meet you at Nashville— a safe trip to you.**

The deposit of ova by the beef bot-fly and the development of warbles or maggots along the spinal region may be treated by having these parts smeared with an ointment of one part of carbolic acid and twenty-five of lard, or a solution of train-oil and black sulphur applied twice in a season is said to be a good precautionary measure.

Veterinarian Butters, of England, recommends careful applications of a liniment of equal parts of carbolic acid, linseed-oil, and spirits of turpentine for the removal of loose folds of skin after operation for removal of capped elbows and in similar conditions of capped hocks when non-inflammatory in character.

Dr. M. E. Knowles, of Montana, reports the spaying of thirty fillies without a single loss. He believes this to be one of the best solutions for controlling the overproduction of horses in the range country.

N. V. Gueorguivsky, a Russian military surgeon, strongly recommends compresses dipped in chemically-pure 2 per cent. solution of bicarbonate of soda for arresting the production of pus and control of inflammation, as superior to the antiseptic agents usually employed, such as carbolic acid solution or iodoform, etc.—*Therapeutic Gazette.*

Dogs are rendered insensible to pain by injecting hypodermatically the following: Morphia hydrochlorat, gr. 1; aqua destil., 2 c.c.; and at seat of incision, 3 c.c. of a 2 per cent. solution of cocaine. After finishing the operation, to counteract the morphia, inject atropia, 1 mgm.; aqua, 2 c.c.

For skin eruptions and ear canker in the dog, ichthyol and glycerin, equal parts, or ichthyol 1 part, alcohol (95 per cent.) 10 parts, and water 10 parts.

